# Fourteen-year analysis of rugby injuries in a pediatric emergency department

**DOI:** 10.1186/s13052-025-02083-8

**Published:** 2025-07-11

**Authors:** Veronica Baioccato, Silvia Paronuzzi, Marco Bazo, Emanuele Zanardo, Viola Sattin, Giulia Lorenzoni, Dario Gregori, Andrea Ermolao, Silvia Bressan

**Affiliations:** 1 Sports and Exercise Medicine Division, University Hospital of Padova, Regional Centre for Exercise Prescription in Chronic Diseases, Padua, Italy; 2 Department of Women’s and Children’s Health, University of Padova, Padua, Italy; 3 Unit of Biostatistics, Epidemiology, and Public Health, Department of Cardiac, Thoracic, Vascular Sciences and Public Health, University of Padova, Padua, Italy

**Keywords:** Rugby, Epidemiology, Emergency, Sports related injuries, Concussion

## Abstract

**Background:**

Rugby is the third most played contact team sport worldwide. Its growing popularity is raising concerns about injuries rates, given its collision-based nature and minimal use of protective gear. However, research on rugby injuries in Italy, especially in the pediatric age group, remains limited.

**Methods:**

This retrospective study examined the visits of patients with rugby-related injuries presenting to a tertiary-care Pediatric Emergency Department (PED) in the Veneto region of Italy, which is the Italian region with the highest rate of youth rugby participation.

**Results:**

The data of 928 PED admissions for rugby-related injuries were extracted from the electronic PED medical records between 2007 and 2021, and analyzed for demographics, injury characteristics and outcomes. Rugby-related injuries accounted for 0.3% of total PED visits (total admissions: 306,508). Injuries to the upper limbs were the most common (40.8%), with contusions being the most frequent diagnoses (34.7%). Concussions accounted for 12.6% of all injuries. The most common injury mechanism was contact with another player (71%). Most injuries were minor, resulting in discharge to home in 97% of cases, while only 3% required hospitalization.

**Conclusions:**

This study provides insights into pediatric rugby injuries in the Italian setting. Our findings highlight the importance of prevention strategies for concussion and upper limb injuries. Further research is warranted to adopt evidence-based interventions for reducing the burden of rugby-related injuries in children and youth.

## Background

Rugby Union (rugby) is the third most popular contact team sport worldwide. Recently, its appeal has been growing consistently amongst the public, with an expected increase in the number of participants over the next few years [1]. Therefore, it can be expected that with the increase in the number of players, there would be a corresponding rise in the incidence of injuries. Previous studies have shown that rugby carries a substantial rate of associated injuries in professional youth and adults athletes [2–4]. One reason could be that rugby is a collision sport lacking mandatory protective equipment, unlike other full-contact sports such as American football and ice hockey [5, 6]. However, over the last few years, injury prevention strategies have improved, including regulation changes, such as the introduction of lower tackles, and the management of contact load, with the attempt of decreasing the incidence of concussion, which is one of the most common rugby-related injury [7].

Regarding pediatric injuries, in particular in Italy, epidemiological data are still limited, and available data are difficult to compare, since the definition of sport injuries used in different surveys often varies and differs across age groups [8, 9]. To standardize injuries classification and facilitate comparison between studies, the International Olympic Committee has published a consensus statement outlining methods for recording and reporting epidemiological data on injuries and illnesses in sports [10, 11].

For this reason, understanding the burden of rugby related injuries in pediatric age is not straightforward, however, according to a recent meta-analysis, approximately 28% of children and adolescent rugby players - engaged in a rugby match-playing season – are likely to sustain injuries [12]. Similarly to reports from other sports, there is a clear trend of increased injury incidence with age [13]: the probability of a player sustaining an injury over a season appears to lie between 9%, for the under 9–12 years age group, and 98%, for the under 18 age group [12, 13]. Of all injuries, concussion represents one of the most frequent diagnoses [9] and its consequences appear to be worse in youths, in terms of time to return to play and symptoms duration. This poses significant safety concerns for the practice of contact sports, particularly during the young age [7].

Based on these considerations, our study aims to explore the epidemiology of rugby-related pediatric injuries, within the Italian geographical context with the highest rate of youth rugby participation. There is a paucity of studies conducted, both in our country and worldwide, on rugby-related injuries, particularly among vulnerable age groups such as pediatric populations [14].

## Methods

This is an observational retrospective study based on Pediatric Emergency Department (PED) visits of children presenting for rugby-related injuries to the University Hospital of Padua between January 1, 2007, and December 31, 2021. Data were extracted from the electronic PED medical records database. PED records including the word “rugby” and all its possible misspelling (ragby, regby, rugbi, and ragbi) in the “history” and “diagnosis” fields were selected, to decrease the risk of omission of relevant records. Retrieved records were reviewed to ensure they were relevant to the objective of the study. Records were included if they corresponded to PED presentations for rugby-related injuries that occurred during both organized and unorganized rugby practice. Records were excluded if:

- the injury was not directly related to playing rugby, such as falls that occurred in the dressing room before, during or after training or the match.
- involved non-traumatic events that occurred during rugby training and/or matches (e.g. non-traumatic headache, non-traumatic abdominal pain)
- were related to planned PED medical checks following the first consultation in the ED, to avoid redundancy of the selected data.

Anonymized data fields were provided by the hospital electronic medical record management team and relevant information was collected through a standardized process, according to a Clinical Report Form developed by the research team. This form included the following data:

- General information: sex, age, year of ED visit, triage urgency category.
- History: body area of injury (upper limbs - shoulder, arm, elbow, wrist, hand; head and neck; trunk; genitals and lower limbs - thigh, knee, leg, ankle, foot); symptoms (e.g. pain, abnormal behavior, limited mobility, any other symptoms reported); head trauma-related symptoms (headache, post-traumatic amnesia, loss of consciousness, headache, vomiting and its features, dizziness); injury mechanism (contact with the ball, with the ground, with opponent, teammate, or other mechanisms).
- Outcome: discharge to home, hospitalization (length of stay in days), surgical treatments, final diagnosis.

Data on injury characteristics were extracted from “history”, “physical examination” and “final diagnosis” data fields during review of medical charts. Based on the information extracted injuries were classified in more detailed groups, by three members of the research team, according to the International Olympic Committee consensus statement for injury sport classification [10].

### Data analysis

Categorical data were reported as frequencies and percentages. Continuous variables were analysed with a Shapiro–Wilk test to assess the normality of the distributions. Given their normal distribution continuous data were reported as means ± standard deviations (SD). Statistical analyses were performed using IBM SPSS software version 26.

Yearly counts of head and neck injuries were first plotted to visually explore potential temporal patterns. Scatterplots with smoothed trend lines, obtained using locally estimated scatterplot smoothing (LOESS), were used to aid interpretation. To formally assess trends, Poisson regression models were fitted separately for total visits, head and neck injuries, and upper limb injuries, with calendar year as a continuous independent variable. Exponentiated coefficients were reported as incidence rate ratios (IRRs) with 95% confidence intervals, representing the average annual relative change in visit counts. The research was approved by the Ethics Committee of the Hospital of Padua with Protocol No. 0053593.

## Results

The initial dataset of all ED accesses, from the 1 st January 2007 to the 31 st December 2021, included 306,508 records. After screening, 928 records were retained. On average, there has been an incidence of 62 injuries per year, with patients mean age of 11.31 ± 2.22 years. Rugby-related pediatric PED admissions accounted for 0.3% of total ED admissions during the study period. A high male prevalence was observed in the study population, with 95.8% male and 4.2% female patients.

Most injuries were non-severe, with 97% of patients discharged and only 3% requiring hospitalization. Most patients visit the PED within 12 h of the injury (53.4%) while only 11.5% presented more than 48 h after the injury had occurred. In more than half of admissions, the triage urgency category assigned was semi-urgent (61.9%), followed by urgent (32.8%), whereas the emergent triage category included only 4 cases (0.4%) (Table 1).

**Table 1:** Characteristics of patient admitted to the pediatric ED for rugby-related injuries

Total number of patients	928
Sex (M); (n, %)	889 (95.8%)
Age (mean ± SD)	11.31 ± 2.22
Time from injury to ED visit (n, %) < 12 h 12-48 h > 48 h Not specified	496 (53.4%) 216 (23.3%) 106(11.4%) 110 (11.9%)
Triage category (n, %) Semi-Urgent Urgent Emergent Not Specified	575(61.9%) 303(32.8%) 4 (0.4%) 46 (4.9%)
ED visit outcome (n, %) Discharge Hospitalization	897 (96.7%) 31(3.3%)

With respect to injury site, the body regions most frequently affected were the upper limbs (40.8%) followed by the head and neck (31.1%), lower limbs (19.2%) and trunk (7.7%). Pooling the data into narrower categories, the most frequently injured area was the head (23.7%), followed by the hand (18.9%), and shoulder (12.4%). Further details are provided in Table 2.

**Table 2:** Distribution of injuries by location and final diagnosis at discharge, according to Orchard Sports Injury and Illness Classification System (OSIICS). The diagnoses at discharge listed in the OSIICS classification but not represented in this table have a frequency of zero cases in our database. Vascular trauma and stump injury have been erased from the table, due the absence of cases. NA: not applicable

			Muscle injury	Muscle contusion	Tendinopathy	Tendon rupture	Brain & spinal cord injury	Peripheral nerve Injury	Fracture	Physis injury	Joint sprain (ligament tear or acute instability episode)	Contusion	Laceration	Abrasion	Organ trauma	Injury without tissue type specified	Total for injury location	Total for macro area
Head and neck	Head		NA	NA	NA	NA	120	0	16	NA	3	49	27	12	2	2	231(23.7%)	302(31.1%)
	Neck		0	0	0	0	1	0	0	NA	48	20	0	1	0	1	71(7.3%)	
Upper limb	Shoulder		0	1	0	0	NA	0	49	0	25	42	0	0	1	2	120(12.4%)	396(40.8%)
	Upper arm		0	0	0	0	NA	0	9	0	1	7	0	0	0	0	17(1.8%)	
	Elbow		0	0	0	0	NA	1	4	0	2	6	0	0	0	0	13(1.3%)	
	Forearm		0	1	0	0	NA	0	9	0	0	4	0	0	0	0	14(1.4%)	
	Wrist		0	0	0	0	NA	0	21	0	13	14	0	0	0	0	48(5.0%)	
	Hand		0	0	0	1	NA	0	68	0	53	55	0	1	0	6	184(18.9%)	
Trunk	Chest		0	0	0	0	0	0	3	0	1	32	0	0	0	1	37(3.8%)	75(7,7%)
	Thoracic spine		0	0	0	0	0	0	0	0	1	5	0	0	0	0	6(0.6%)	
	Lumbosacral		0	2	0	0	1	0	0	0	1	8	1	0	0	0	13(1.3%)	
	Abdomen		0	0	0	NA	NA	0	0	NA	1	15	0	0	2	0	19(2.0%)	
Lower limb	Hip/groin		0	1	1	0	NA	0	0	0	1	6	0	0	0	0	9(0.9%)	186(19,2%)
	Thigh		1	1	0	0	NA	0	2	0	0	9	0	0	0	0	13(1.3%)	
	Knee		1	0	0	0	NA	0	3	1	31	23	3	3	0	3	68(7.0%)	
	Lower leg		0	0	0	0	NA	0	7	1	0	4	1	0	0	1	14(1.4%)	
	Ankle		0	0	0	0	NA	0	6	1	35	11	0	0	0	1	54(5.6%)	
	Foot		0	0	0	0	NA	0	5	0	3	20	0	0	0	0	28(2.9%)	
Unspecified	Region unspecified		0	0	0	0	NA	0	0	0	3	6	0	0	0	2	11(1.1%)	11(1.1%)
Multiple regions	Single injury crossing two or more regions		0	0	0	0	0	0	0	0	0	1	0	0	0	0	1(0.1%)	1(0.1%)
	Total		2(0.2%)	6(0.6%)	1(0.1%)	1(0.1%)	122(12.6%)	1(0.1%)	202(20.5%)	3(0.3%)	222(22.9%)	337(34.7%)	32(3.3%)	17(1.8%)	5(0.5%)	19(1.9%)	971	971

The most frequent diagnosis upon discharge was contusion (34.7%), followed by joint sprain (22.9%), fracture (20.9%), concussion (12.6%), and lower percentages for other types of injuries, including one case of spleen rupture (see Table 2).

Injury mechanisms are detailed in Table 3, reported in a separate section for concussion diagnoses compared to all other types of injuries. The most common mechanisms are the same for both subgroups, but with different percentages: contact with a teammate was the most frequent, followed by contact with an opposing player.

**Table 3:** Distribution of injury mechanisms for all types of injury and concussion: the total number of injury mechanisms across all categories is greater than the number of patients due to some patients reporting multiple injuries

Mechanism of injury		
	All injuries except concussion (n)	Concussion (n)
Contact with a teammate	309 (36.4%)	58 (47.9%)
Contact with a player of the opponent team	108 (12.7%)	26 (21.5%)
Contact with the ball	30 (3.5%)	0
Contact with the ground	106 (12.5%)	19 (15.7%)
Other or not specified	297 (34.9%)	18 (14.9%)
Total	850	121

Peak admissions to the pediatric ED occurred in 2013 (11%), while 2020 and 2021 had lower number of admissions due to COVID-19 restrictions.

**Fig. 1:**
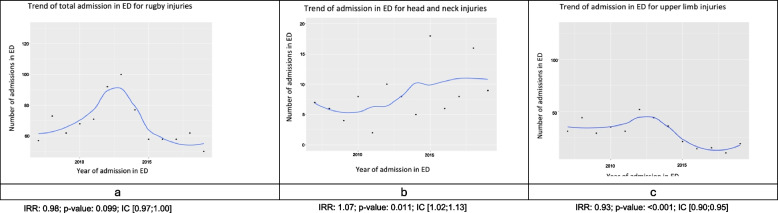
Temporal trend for: **a** total admissions for total rugby injuries to the PED; **b** admissions for head/neck injuries; and **c** admissions for upper limb injuries

The plot of rugby-related PED visits until 2019 are shown in Fig. 1a. Years 2020 and 2021 were excluded due to the potential bias caused by the coronavirus pandemic. Separate analyses were conducted for head and neck injuries (Fig. 1b) as well as upper limb injuries (Fig. 1c). The incidence rate ratios (IRRs) and their 95% confidence intervals are reported at the bottom of each graph.

## Discussion

To the best of our knowledge, this study is the first to analyze pediatric rugby-related injuries from an Italian emergency department. This report gives a useful insight for pediatricians and sports and exercise medicine health care professionals, that are involved in the care of youth rugby injuries in Italy.

Although the number of studies on injury epidemiology in rugby has increased in the last few years, evidence remains scarce, mainly due to the heterogeneity of data collection methodology and research settings. Nonetheless, the results of our data analysis align closely with those reported in the literature [14].

One of the main findings of our study, is that the upper limb is the most frequent injured body region, consistent with findings from several other studies [9, 15–17]. The second most common site of injury varies across these studies; however, the head seems to be more frequently injured than lower limbs, in line with our findings [15, 16]. It must be noted that several studies analyzing injuries in different settings, and sometimes using different injury classifications, found different results [17, 18]. However, when focusing on studies conducted in the ED setting and including pediatric age groups, results are consistent across the USA, Ireland, Australia and Scotland [8, 17, 19, 20].

An important aspect is the mechanism by which injuries occur. The literature consistently identifies contact with another player as the most frequent mechanism [8, 17]. This is also confirmed by our data; specifically, contact with a teammate appears to be more frequently the cause of injury than contact with an opponent player. Other studies have also analyzed which component of the play is the most frequently associated with injuries, and it has been found that the tackle is definitely the most dangerous [8, 15, 19, 20].

According to the Orchard Sports Injury and Illness Classification System (OSIICS) classification, we found that the most frequents type of injuries were contusions (34,7%), followed by joint sprains (22.9%), fractures (20.9%) and concussions (12.6%). One of the main reasons for the different percentages of injury sites reported in the literature, could be that rugby is a sport where rules, gameplay, and the utilization of personal protective gear differ depending on age groups [10, 18]. For instance, rules of the scrum vary across different age categories and are introduced at different ages in various countries [20]. However, contusions, fractures, sprains and concussions consistently rank as the top four injuries in every study [12, 14–16].

As for the severity of injury, our study aligns with the literature, with 97% of injuries managed acutely in the ED and only 3% requiring hospitalization [17].

Special attention should be given to concussions, which have a prevalence of 12.6% in our study, similar to what reported by McIntosh et al. [21]. However, incidence rates vary dramatically across studies due to the difference in injury definition, lack of objective diagnosis criteria, different levels of competition and gender-related aspects [22]. Kirkwood et al. conducted a metanalysis in 2015 and found out that the incidence of concussion in youth rugby union ranged from 0.2 to 6.9 concussions per 1000 player-hours, equivalent to a probability for a player of sustaining a concussion over a season between 0.3% and 11.4% [23].

Our study spans across a period that includes the three years prior the rule "recognize and remove" implemented in 2012, was introduced [24, 25]. This strategy involves identifying signs of concussion or head injury and immediately removing the affected player from the game for further assessment with the aim of prioritizing player safety. This, coupled with limited awareness of the issue, may have led to a lower recognition of concussion and thus its underdiagnosis.

However, the incidence of concussion remains high, needing careful attention, especially for the potential serious long-term consequences. For this reason, several studies have been conducted to investigate possible strategies to reduce the burden of this type of injury. One of the most discussed proposals for reducing the incidence of concussions in rugby is lowering the height of the tackle. This measure is based on the assumption that reducing the point of contact during a tackle may decrease the risk of head impact and, consequently, the likelihood of concussions. However, both van Tonder and Stokes did not find any statistically significant reduction of concussion’s incidence by reducing the tackle height from the line of the shoulder to the armpit of the ball carrier, whether in amateur or professional settings [26, 27]. In detail, Van Tonder’s results showed a reduction in the incidence of concussion, which was not statistically significant but may be clinically relevant; while Stokes et al. found a lack of reduction in the incidence of this injury, due to the change of tackling technique that apparently makes the tackler more vulnerable. Understanding impacts in rugby is crucial for injury prevention strategies. A comprehensive approach to injury prevention may require combining tackle height adjustments with educational programs focusing on safe tackling techniques, improved referee enforcement, and continued monitoring of injury patterns [26, 27]. Moreover, a recent systematic review suggests that the most effective primary prevention strategy for sport-related injuries (such as concussions and hamstrings lesions) appears to be neuromuscular training, followed by education, changes in law and use of specific equipment [7]. Further research is needed to understand the effectiveness of these measures, which could play a crucial role in reducing the incidence of this type of injuries. Additionally, it is essential to emphasize the importance of raising awareness among players, coaches, and medical personnel regarding the recognition, assessment, and management of concussions to minimize consequences as much as possible. As expected, data from adolescent rugby players, suggest that coaches were less able to recognize symptoms of concussion compared with health care professionals, probably because of lack of concussion education, and that this could lead to an underestimation of the incidence of concussion in lower levels of play, emphasizing the need of training the team personnel [22, 28]. Also, the knowledge that the tackle is responsible for more concussions, than any other contact event, suggests that interventions that focus on tackle technique training, law change (e.g., changes to the legal tackle height if proven effective) and player preparation, may be more effective at reducing injury risk than other interventions in this setting [18].

Poisson regressions analysis were performed to examine trends over time. The trend of total visits shows a peak in 2013, followed by a rapid decline and stabilization in the subsequent years, with the lowest value reached in 2019. Given that the data pertains only to the pediatric emergency department of Padua, it is difficult to determine whether any specific cause or event is responsible for this trend. There are not much data in the literature, especially in Italy. However, England, Scotland and Australia professional rugby injury surveillance programs found that the incidence of injuries is stable in the same time period [25]. Conversely, evidence from US emergency departments from 2004 to 2013 found an increased incidence of injuries, in the same period [17].

Trend for head and neck injuries shows a seemingly slight upward progression, which, however, appears to be too modest to be attributed to an increased awareness of concussions after 2012, year of introduction of recognize and remove protocol. Published data confirm an increase in the diagnosis of concussions, their severity and burden. The Scotland injury program confirmed a growth of concussion diagnosis between 2009-2016 and a deflection after that [25, 29].

Finally, the trend for PED admissions due to upper limb injuries appears to be decreasing over time. While it is challenging to pinpoint a specific underlying cause, one possibility might be that the management of some of these injuries has improved or has been encouraged to take place in the field, outside the hospital, after a world rugby medical education program was implemented [30].

Our study has several limitations. First, it is a retrospective study. Therefore, ED histories could have some missing information (i.e. mechanism of injury or game phase). Indeed, the database records only a brief case narrative for each injury, and mechanisms of injury could not be determined in 22% of cases.

Another limit in this study is the lack of female athletes, probably due to the low representation in the local rugby union. Nevertheless, the number of female participants has been steadily increasing over the past few years, hence providing us with a potentially more significant sample to include in future analysis [31].

It is important to underline that not all rugby injuries are equally reported to the ED. Many injuries are managed at alternative healthcare facilities, outpatient sport medicine practices or by team doctors/physiotherapists. Consequently, this could lead to a bias in the data towards more severe injuries and might result in an underrepresentation of certain types of milder injuries.

Furthermore, our study includes data from the years 2020-2021, during which the data might be skewed or altered by COVID-related restrictions.

## Conclusion

This study aligns with existing literature on the epidemiology of rugby injuries in pediatric age, conducted in similar setting, emphasizing how the primary injury mechanism is contact with another player causing most often soft tissues injuries. It also highlights that the head and upper limbs are the most involved areas, suggesting that injury prevention strategies, should focus on these body sites. The high prevalence of concussion reported confirms the need for special attention to its recognition and provision of proper advice upon discharge from the PED. Lastly, the slightly increasing trend for concussion suggests that adequate levels of awareness have not yet been reached in our country, if compared with the literature. Education programs are useful tool for recognition and management of injuries, especially when translated in the local language. This report provides a useful insight for pediatricians and sports and exercise medicine health care professionals involved in the care of youth rugby injuries in Italy.

## Data Availability

The datasets used and/or analyzed during the current study are available from the corresponding author on reasonable request.
